# Engineering Non-cellulosic Polysaccharides of Wood for the Biorefinery

**DOI:** 10.3389/fpls.2018.01537

**Published:** 2018-10-23

**Authors:** Evgeniy Donev, Madhavi Latha Gandla, Leif J. Jönsson, Ewa J. Mellerowicz

**Affiliations:** ^1^Department of Forest Genetics and Plant Physiology, Swedish University of Agricultural Sciences, Umeå, Sweden; ^2^Department of Chemistry, Umeå University, Umeå, Sweden

**Keywords:** non-cellulosic polysaccharides, woody biomass, secondary cell wall, hemicellulose, pectin, galactan, tree genetic improvement, wood biorefining

## Abstract

Non-cellulosic polysaccharides constitute approximately one third of usable woody biomass for human exploitation. In contrast to cellulose, these substances are composed of several different types of unit monosaccharides and their backbones are substituted by various groups. Their structural diversity and recent examples of their modification in transgenic plants and mutants suggest they can be targeted for improving wood-processing properties, thereby facilitating conversion of wood in a biorefinery setting. Critical knowledge on their structure-function relationship is slowly emerging, although our understanding of molecular interactions responsible for observed phenomena is still incomplete. This review: (1) provides an overview of structural features of major non-cellulosic polysaccharides of wood, (2) describes the fate of non-cellulosic polysaccharides during biorefinery processing, (3) shows how the non-cellulosic polysaccharides impact lignocellulose processing focused on yields of either sugars or polymers, and (4) discusses outlooks for the improvement of tree species for biorefinery by modifying the structure of non-cellulosic polysaccharides.

## Non-Cellulosic Polysaccharides in Wood

Plant cell walls constitute the most abundant renewable resource on Earth (Pauly and Keegstra, 2008). Wood, which essentially consists of cell walls, is naturally degradable and renewable, and technologies are currently being developed aiming at utilization of all organic wood components, i.e., cellulose, lignin, non-cellulosic polysaccharides, and extractives. Non-cellulosic polysaccharides, which include hemicelluloses, pectins, type II arabinogalactan (AG-II), and callose, account for roughly one third of wood dry weight. Among these four groups, hemicelluloses are most abundant contributing to 26–33% of the dry weight in softwoods (conifer species) and 19–34% in hardwoods (dicotyledonous species) depending on species, cell type, developmental stage, and environmental conditions (Sjöström, 1993).

Non-cellulosic polysaccharides exhibit remarkable variability in different layers of wood cell walls, and chiefly define these layers (Mellerowicz and Gorshkova, 2012). Thus, the middle lamella is dominated by pectins, the primary cell wall (PCW) layer by pectins and xyloglucan, the secondary cell wall (SCW) layers by xylans and mannans, and the gelatinous layer (G-layer) present as a tertiary layer in tension wood fibers by galactans and AG-II. These different polysaccharides blend with the lignin matrix and cellulose microfibrils, and are involved in covalent, ionic, and hydrophobic interactions with other cell wall components and with themselves (Cosgrove, 2005; Scheller and Ulvskov, 2010). They are the main source of lignin carbohydrate complexes (LCCs) linking to lignin by ether, glycoside, or ester bonds (Lawoko and Henriksson, 2006; Balakshin et al., 2007; Giummarella and Lawoko, 2016; Giummarella et al., 2016). Thereby, the non-cellulosic polysaccharides affect cell wall architecture, wood traits, and properties of lignocellulosic biomass being favorite targets for improving biomass properties (reviewed in Tavares et al., 2015; Damm et al., 2016; Marriott et al., 2016; Wang et al., 2016; Smith et al., 2017). They are also a precious source of large amounts of assimilated carbon for which clever applications are being sought (e.g., Zhao et al., 2015; Oinonen et al., 2016).

**FIGURE 1:**
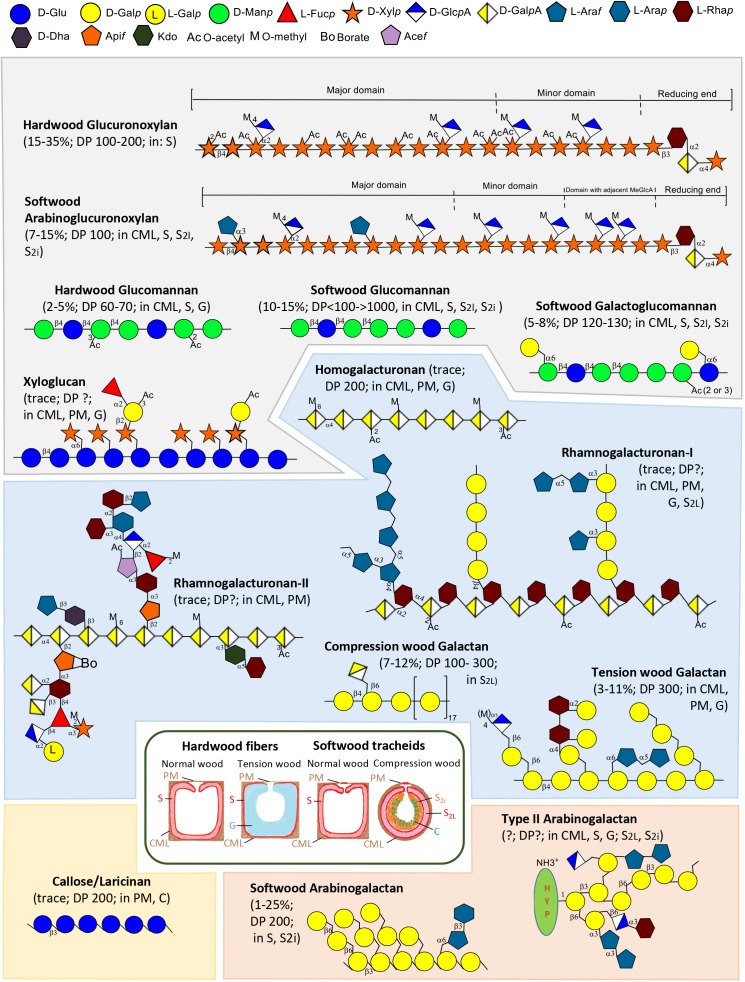
Schematic illustration of types of non-cellulosic polysaccharides of wood, including hemicelluloses (gray background), pectins (blue background), callose (yellow background) and AGs-II (orange background), and hardwood fibers and softwood tracheids (inset). Polymer structures were based on different sources: hardwood GX (Teleman, 2009; Smith et al., 2017), softwood arabinoglucuronoxylan (Teleman, 2009; Martínez-Abad et al., 2017; Smith et al., 2017), hardwood and softwood glucomannan (GM), softwood GGM, tension and compression wood galactans, callose (Teleman, 2009), xyloglucan (Carpita and McCann, 2000; Teleman, 2009), HG (Atmodjo et al., 2013), RG-I and -II (Edashige and Ishii, 1996, 1997, 1998; Atmodjo et al., 2013), AG-II (Carpita and McCann, 2000; Hijazi et al., 2014), softwood arabinogalactan (Ponder and Richards, 1997; Teleman, 2009). Polymer localization is based on the following sources: hardwood GX and mannans (Kim and Daniel, 2012; Gorshkova et al., 2015; Guedes et al., 2017), softwood arabinoglucuronoxylan (Altaner et al., 2010; Donaldson and Knox, 2012), callose (Altaner et al., 2010; Zhang et al., 2016), xyloglucan (Bourquin et al., 2002; Sandquist et al., 2010; Nishikubo et al., 2011; Donaldson and Knox, 2012; Kim and Daniel, 2013; Guedes et al., 2017), HG (Kim and Daniel, 2013), RG-I/compression wood galactan/tension wood galactan (Gorshkova et al., 2015; Zhang et al., 2016; Guedes et al., 2017), AG-II/softwood arabinogalactan (Altaner et al., 2010; Guedes et al., 2017). PM, pit membrane; CML, compound middle lamella; S, secondary wall layer (S-layer), G, gelatinous layer (G-layer); C, cavities; S_2i_, inner S_2_ layer; S_2L_, outer lignified S_2_ layer.

Among hemicelluloses, xylan, which includes the glucuronoxylan (GX) of hardwoods and the arabinoglucuronoxylan of softwoods (Figure 1), is a ubiquitous component of wood SCWs (Donaldson and Knox, 2012; Kim and Daniel, 2012). Approx. 60% of the xylopyranosyl residue (Xyl*p*) of hardwood xylan are mono- or di-acetylated (Teleman et al., 2000, 2002). The acetyl groups compete with glucuronic acid for Xyl*p* position 2, and a decrease of one of these substituents usually leads to an increase of the other (Chong et al., 2014; Lee et al., 2014). Mannans, which include water-soluble galactoglucomannan (GGM) and water-insoluble glucomannan (GM) (Figure 1), are the most abundant hemicelluloses in softwood SCWs, whereas hardwood SCWs contain lower fractions of GM (Teleman, 2009). Xyloglucan (Figure 1) is localized in PCWs of hardwoods and softwoods (Bourquin et al., 2002; Donaldson and Knox, 2012; Kim and Daniel, 2013), where it may associate with hydrophobic cellulose surfaces or become entrapped inside cellulose fibrils (Park and Cosgrove, 2015).

Pectins, which include homogalacturonan (HG), rhamnogalacturonan I (RG-I), and rhamnogalacturonan II (RG-II) (Figure 1), are acidic polysaccharides. They constitute a large part of the middle lamella and PCW layers, jointly referred to as the compound middle lamella (CML) (Kim and Daniel, 2013). Reaction wood, such as tension wood of hardwood and compression wood of softwood, typically contains high mass fractions of β-1,4-galactans (Figure 1) presumably associated with RG-I.

Water-soluble softwood arabinogalactan, a variant of AG-II (Figure 1), is highly abundant in larch (25%). Other softwoods and hardwoods contain small amounts of AG-II. AG-II may be covalently linked to xylan and pectin (Tan et al., 2013). Callose (Figure 1), or laricinan, accumulates in hardwoods and softwoods in response to damage and stress (Teleman, 2009). It is abundant in pits and between cavities of the inner S2 layer in compression wood (Hoffman and Timell, 1970; Chaffey and Barlow, 2002; Altaner et al., 2010; Zhang et al., 2016).

This review addresses the importance of the non-cellulosic polysaccharides in technological processes currently used in wood biorefining, and prospects of altering them in trees for obtaining either higher productivity or improved lignocellulose properties, like extractability or improved biochemical conversion to sugars.

## Fate of Non-Cellulosic Polysaccharides During Wood Biorefining

Biorefining of wood includes the pulping (mechanical and chemical pulping, as well as combinations thereof), biochemical processes, and thermochemical processes. Mechanical pulping aims at high recovery of all major wood constituents, including non-cellulosic polysaccharides (Sjöström, 1993; Ek et al., 2009). In contrast, chemical pulping and subsequent bleaching steps are designed to target the lignin and preserve the cellulose, whereas the fate of the non-cellulosic polysaccharides is strongly dependent on the aim and the process technology, which can be Kraft (sulfate), sulfite, soda, or organosolv pulping (Sjöström, 1993; Ek et al., 2009). For example, dissolving pulp manufacture based on sulfite pulping, or, sometimes, Kraft pulping, aims at producing a cellulose of relatively high purity, which means that most of the non-cellulosic polysaccharides are degraded and removed together with the lignin. In many other processes, such as conventional Kraft pulping for manufacturing of paper products, preservation of hemicelluloses is beneficial, as the pulp yield will then be higher.

Biochemical conversion is typically based on saccharification of the polysaccharides using pretreatment and enzymatic hydrolysis. This creates monosaccharides, which are then refined further using microbial fermentation or chemical catalysis. The aim of the pretreatment is to make the cellulose susceptible to enzymatic hydrolysis, which otherwise would be too slow and provide too low sugar yields. Among many different pretreatment methods (Yang and Wyman, 2008; Sun et al., 2016; Jönsson and Martín, 2016), hydrothermal pretreatment under acidic conditions, with or without externally added acid and with or without the disruptive effect of steam explosion, is a common approach. Due to autohydrolysis and formation of carboxylic acids, which are derived mainly from the non-cellulosic polysaccharides, the process will be acidic even without externally added acid (Jönsson and Martín, 2016). The main target of hydrothermal pretreatment under acidic conditions is the hemicellulose. Cellulose and lignin are also affected, but typically to much lesser degree than hemicelluloses, which can be degraded almost quantitatively in well-optimized pretreatment (Wang et al., 2018). The severity of the hydrothermal pretreatment (time, temperature, and acidity) needs to be adapted to the feedstock. Softwoods require higher severity, whereas hardwoods can be processed using lower severity. Nevertheless, pressurized reactors and temperatures in the range 160–240°C (Sun et al., 2016) are typically used to create a pretreated material that is suitable for subsequent enzymatic saccharification.

Thermochemical conversion processes, such as combustion, gasification, and pyrolysis, will degrade all organic wood constituents and are less relevant within the context of the current review.

## Role of Non-Cellulosic Polysaccharides in Recalcitrance and Attempts to Improve Convertibility

### Improvement of Xylan Structure

#### Xylan Content and Length Affect Saccharification and Plant Growth

Xylan is a key factor of recalcitrance, mainly by reducing cellulose accessibility (Bura et al., 2009; De Martini et al., 2013), prompting efforts to reduce its content in hardwoods. Attempts have been made using *Arabidopsis* as a model, either by targeting the xylan synthase complex or the enzymes synthesizing the reducing end sequence (RES) (Smith et al., 2017). However, strong reductions in xylan content led to mechanical failure of vessels [the so-called irregular xylem phenotype (IRX)] and stem weakening. Subsequent work with *Populus* indicated that xylan content can be reduced by 5–50% by knocking down (KD) different xylan biosynthetic genes including *GT47C* (Lee et al., 2009), two *GAUT12/GT8D* paralogs (Lee et al., 2011; Li et al., 2011; Biswal et al., 2015), *GT43A* and *GT43B* (Lee et al., 2011), and *GT43B* along with *GT43C* clade genes (Ratke et al., 2018) or by expressing fungal xylanase *HvXyl1* and targeting it to cell walls (Kaida et al., 2009; Table 1). Such reductions either did not affect (Lee et al., 2009) or stimulated growth (Biswal et al., 2015; Ratke et al., 2018), decreased xylem cell wall thickness (Li et al., 2011; Ratke et al., 2018), which sometimes (Lee et al., 2009, 2011) was coupled to a mild IRX phenotype. Beside xylan, the cellulose content decreased in case of *GT47C* KD, coupled with increased lignification (Lee et al., 2009). A similar increase in lignin coupled with brittleness of stems was observed in strong KD *GAUT12-1* and *-2* (Li et al., 2011), but not when only *GAUT12-1* was reduced (Biswal et al., 2015). Lignin syringyl/guaiacyl (S/G) ratio was increased in *GAUT12-1* KD poplar (Biswal et al., 2015), and in severe KD *GT43B* poplar (Lee et al., 2011). However, the S/G ratio was reduced without change in lignin content in mildly affected *GT43B* and *GT43C* KD (Ratke et al., 2018). Thus, it is difficult to predict how lignin might be affected in transgenic lines with reduced xylan content, and these changes should be carefully monitored, since they affect saccharification.

**Table 1:** Effects of engineering expression of genes encoding proteins affecting non-cellulosic polysaccharides on enzymatic saccharification and cell wall composition of woody species.^a^

Gene	Approach	Species	Protein	Protein family	Promoter	Role of protein	Saccharification efficiency	Phenotypic effect	Effect on cell wall composition	Reference
*PtxtPME1*	Down- or up-regulation	Hybrid aspen	Pectin methyl esterase (PME)	CE8	*35S*	Removal of methyl ester groups from HG	EH(+), AEH(-) in down-regulated lines	In down-regulated lines: Height(ND) diameter(+)	Up-regulation resulted in (-) in DM while downregulation resulted in (+) DM and (-) in the amount of galacturonan.	Siedlecka et al., 2008; Escamez et al., 2017
*PoGT47C*	*RNAi* down-regulation	Hybrid poplar	Glycosyl transferase	GT47	*35S*	Probably biosynthesis of RES in X	EH(+)	Growth(ND)	S/G(+), Xyl content(-), Glu content(-), GX(-), thickness of cell walls(-), RES of GX (-)	Lee et al., 2009
*AaXEG2*, *HvXYL1*, *Tv6GAL*, *AtCel1*	Over-expression	Poplar (*P. alba*)	Xyloglucanase, xylanase, galactanase, and glucanase	GH5, GH10, GH35, and GH9	*35S*	Hydrolysis of XG, X, Ga, and GM	EH(+) for *AaXEG2, HvXYL1* and *AtCel1*, EH(-) for *Tv6GAL*	Growth(+) for *AaXEG2*.	L content (ND) for *HvXYL1* and *AtCel1*, (-) for *AaXEG2*.	Park et al., 2004; Kaida et al., 2009
*AaXEG2*	Over-expression	*Acacia mangium*	Xyloglucanase	GH5	*35S*	Hydrolysis of XG	EH(+)	Growth(+)	Cellulose(+), L(-),wall bound XG in the xylem(-)	Hartati et al., 2011; Kaku et al., 2011
*PtxtXET16A/PtxtXTH34*	Up-regulation	Hybrid aspen	Xyloglucan endotransglucosylase (XET)	GH16	*35S*	Hydrolysis and re-ligation of XG	EH(ND), AEH(ND)	Growth(ND)	Loosely bound XG fraction(-), tightly bound XG fraction(+), PCW XG(+)	Nishikubo et al., 2011; Escamez et al., 2017
*PoGT43B, PtxtGT43B and PtxtGT43C*	RNAi down-regulation	Poplar *(P. trichocarpa), hybrid aspen*	Glycosyl transferases	GT43	*35S* and *GT43B*	Scaffolding in X synthase complex, X backbone biosynthesis	EH(+), AEH(ND)	Growth(+) for *GT43B* promoter	Xyl content relative to RES (Xyl:RES) in X(-). Cell wall thickness(-), wood density(-), MFA(-), S/G(-).	Lee et al., 2011; Ratke et al., 2018
*PtxtPL1-27*	Up-regulation	Hybrid aspen	Pectate lyase (PEL)	PL1	*35S*	Cleavage of HG backbone	EH(+) (for release of Xyl), AEH(+)	Growth(-)	L(+), M(-), TFA soluble Glu (+), solubility of pectin, X, and XG(+)	Biswal et al., 2014
*PtGAUT12-1/PtGT8D-1, PtGAUT12-2/PtGT8D-2*	RNAi down-regulation	Poplar (*P. trichocarpa, P.deltoides)*	Galacturonosyl transferase	GT8	*35S*	Biosynthesis of pectin and RES in X	EH(+)	Growth(ND or +)	L (+), mechanical strength (-), S/G(+), Xyl(-), GalA(-), pectin(-), X(-), Rha(-)	Lee et al., 2011; Li et al., 2011; Biswal et al., 2015
*PcGCE*	Over-expression	Hybrid aspen	Glucuronoyl esterase	CE15	35S	Hydrolysis of ester linkages between MeGlcA and L	EH(-), AEH(-), Cellulose conversion (+)	Growth(-)	L(+), C(-), S/G(+), Ara(-), Rha(-), MeGlcA(-). Compositional changes of pectin.	Gandla et al., 2015
*PtxtXyn10A*	Antisense down-regulation	Hybrid aspen	Xylan endotransglycosylase	GH10	35S	Hydrolysis and re-ligation of X chains	EH(ND), AEH(ND)	Growth(+)	Ga(-), X(+), TW content in stem(-), MFA(-)	Derba-Maceluch et al., 2015; Escamez et al., 2017
*PtrDUF579-3*	Antisense down-regulation	Poplar *(P. trichocarpa)*	GX methyl transferase (GXM3)	GT8	35S	Methylation of GlcA in GX biosynthesis	AEH(+)	Growth(ND)	Mechanical strength of stem(-), methylation of GlcA in GX(-)	Song et al., 2016
*PdDUF231A*	Up-regulation	Poplar (*P. deltoides)*	Acyl transferase/esterase	DUF231	UBI3	Probably acetylation of X	EH(+)	Growth(+)	L(-), Cellulose(+),acetylated X at O-3(+)	Yang et al., 2017
*PtxtRWA* (AB and CD clades)	RNAi suppression of RWA family	Hybrid aspen	RWA-A, RWA-B, RWA-C, and RWA-D	RWA	GT43B	Probably transport of acetyl-CoA to Golgi	EH(+), AEH(+)	Growth(ND)	Total acetylation and X acetylation at position O2(-), MeGlcA(+)	Pawar et al., 2017b
*AnAXE1*	Over-expression	Hybrid aspen	Acetylxylan esterase (AX)	CE1	35S	Deacetylation of X and possibly other polymers	EH(+) AEH(+)	Growth(ND)	Total acetylation and acetylation of X at position O2 (-). X chain length and size of L polymer(-). L solubility (+), S/G(-)	Pawar et al., 2017a
*PdGAUT4*	RNAi down-regulation	Poplar *(P. deltoides)*	α-1,4-Galacturonosyl transferase	GT8	35S	Probably synthesis of HG	HWEH(+) (Glu yield)	Growth(+)	S/G(+), HG(-), RGII(-), GalA(-), Calcium and boron in the cell wall(-). HG and RGII cross-link in cell wall(-)	Biswal et al., 2018

*^a^AEH, saccharification efficiency of enzymatic hydrolysis after acid pretreatment; Ara, arabinose; C, carbohydrate; DM, degree of methylation; EH, saccharification efficiency of enzymatic hydrolysis without pretreatment; Ga, galactan; GalA, galacturonic acid; Glu, glucose; GlcA, glucuronic acid; GM, glucomannan; GX, glucuronoxylan; Hybrid aspen, Populus tremula ×tremuloides; HG, homogalacturonan; Hybrid poplar, Populus alba ×tremula; HWEH, saccharification efficiency of enzymatic hydrolysis after hot water pretreatment; L, lignin; M, mannan; MeGlcA, 4-O-methylglucuronic acid; MFA, microfibril angle; ND, No detectable effect compared to WT; RGII, Rhamnogalacturonan II; Rha, rhamnose; RES, reducing end sequence of xylan; S/G, syringyl:guaiacyl ratio; TFA, trifluoroaceticacid; TW, tension wood; X, xylan; Xyl, xylose; XG, xyloglucan; (+), increase compared to WT; (–), decrease compare to WT*.

For non-pretreated wood, downregulation of *GT43* genes resulted in a 30% increase in glucose yield in enzymatic saccharification (Lee et al., 2011; Ratke et al., 2018), but the benefits were less apparent after acid pretreatment (Ratke et al., 2018; Table 1). Reductions in xylan content by downregulation of *GAUT12/GT8D* did not improve saccharification without pretreatment (Lee et al., 2011) or did so only slightly (after steam pretreatment) (Biswal et al., 2015) whereas post-synthetic xylan reduction resulted in approx. 50% increased glucose yield in saccharification after steam pretreatment (Kaida et al., 2009; Table 1). There might be several and possibly opposing factors at play affecting saccharification. For example, cell wall thickness, lignin content and composition, and content of tension wood can all affect glucose yields (Escamez et al., 2017). KD *GAUT12/GT8D* poplar had less LCCs, which contribute to recalcitrance (Min et al., 2014a). Clearly, manipulation of xylan induces indirect effects, some of which, such as increased growth (Biswal et al., 2015; Derba-Maceluch et al., 2015; Yang et al., 2017; Ratke et al., 2018), or increased drought tolerance (Keppler and Showalter, 2010) are interesting for biotechnological applications.

#### Xylan Acetylation Affects Cell Wall Architecture

Deacetylation of lignocellulosic biomass prior to enzymatic saccharification results in improved sugar yields (reviewed by Pawar et al., 2013). For lignocellulosic biomass with high acetyl content such as hardwoods, reduction of acetylation might have an added benefit for ethanolic fermentation processes, as high concentrations of acetic acid are inhibitory to fermenting microorganisms (reviewed by Jönsson and Martín, 2016).

Modest reductions in acetylation in KD *RWA* aspen (Pawar et al., 2017b) and in aspen expressing fungal xylan acetyl esterase *An*AXE1 targeted to cell walls (Pawar et al., 2017a; Table 1) were well tolerated by plants. These plants yielded 20–25% more glucose in enzymatic saccharification without pretreatment. Results with *Arabidopsis* (Pawar et al., 2016) suggested that deacetylation *in planta* reduces recalcitrance by other mechanism than reducing the inhibition of xylan enzymatic hydrolysis by acetyl groups. Indeed, aspen expressing *AnAXE1* exhibited increased lignin solubility and reduced xylan content, xylan chain length, and lignin S/G ratio (Pawar et al., 2017a). Increased extractability of lignin and xylan agrees with the suggested xylan models (Ruel et al., 2006; Busse-Wicher et al., 2014), where the minor xylan domain (Figure 1) interacts with lignin. This domain, characterized by consecutive Xyl*p* acetylation, would become (after deacetylation) more susceptible to hydrolysis by wall-residing enzymes, such as XYN10A (Derba-Maceluch et al., 2015), leading to loosening of xylan fraction interacting with lignin.

Overexpression of *PdDUF231A*, similar to *AtPMR5*, in *P. deltoides* resulted in increased xylan acetylation and surprisingly in improved saccharification without pretreatment (Yang et al., 2017; Table 1). Decreased lignin content and increased cellulose content in transgenic lines might have affected the sugar yield.

#### Significance of Glucuronosylation

Glucuronosylation of xylan makes its backbone less prone to hydrolysis by GH10 and GH11 xylanases, and requires α-glucuronidases for saccharification (Mortimer et al., 2010). It is also associated with LCCs of hardwoods (Min et al., 2014a,b; Bååth et al., 2016). The majority of glucuronate in SCW xylan is methylated (Teleman, 2009) and KD of a GX methyl transferases *DUF579-3*/*GXM3* in poplar reduced not only methylation but also resulted in reduced xylan glucuronosylation, and reduced growth (Song et al., 2016). Xylose yield of acid pretreatment increased as well as glucose yield of enzymatic saccharification. Thus, methylation of glucuronate is a promising target, but some means of avoiding growth penalty need to be designed.

To reduce ester links between GX and lignin in aspen, a fungal glucuronoyl esterase was expressed and targeted to cell walls (Gandla et al., 2015; Table 1). Increased cellulose-to-glucose conversion was observed, but plants exhibited premature leaf senescence and impaired growth (Gandla et al., 2015). There was a decrease in extractives and an increase in lignin (Gandla et al., 2015) and mechanisms underlying these responses are not understood.

### Prospects for Mannan Structure Improvement

GGM is tightly associated with cellulose microfibrils (Schröder et al., 2009; Salmén, 2015) and increasing its extractability could be beneficial for saccharification. Mannan hydrolysis *in planta* by overexpression of plasma-membrane-bound mannanase MAN6 induced production of active galactoglucomanno-oligosaccharides that modified growth of poplar and inhibited SCW formation (Zhao et al., 2013), making this approach problematic. However, overexpression of extracellular GM-active endoglucanase *At*Cel1 in poplar lead to approx. 30% increase in sugar yield and in cellulose conversion in saccharification after steam pretreatment (Kaida et al., 2009).

Mannan biosynthetic genes identified to date include a GM synthase (CSLA clade) (Suzuki et al., 2006), a mannan galactosyltransferase (GT34) (Edwards et al., 2004), and unknown function GT65 members *At*MSR1 and 2 (Wang et al., 2013), and could be employed for biomass modification. For example, increasing the degree of galactosylation of mannan by increasing expression of mannan galactosyltransferase might lead to higher solubility of GGM (Edwards et al., 2004).

### Xyloglucan and Pectins – Minor Wood Components With High Impact on Properties

Several lines of evidence indicate that xyloglucan and pectins, despite being minor wood components (below 5% dry weight), have significant effects on wood properties, including recalcitrance. Increased xyloglucan content in aspen obtained by overexpressing *XTH34/XET16A* did not affect growth (Nishikubo et al., 2011) nor saccharification (Escamez et al., 2017), but its reduction in poplar, achieved by expression of fungal xyloglucanase *AaXEG2*, highly stimulated growth, wood cellulose content, density, and mechanical strength in the greenhouse-grown poplar (Park et al., 2004; Table 1). Cellulose microfibrils were larger in transgenic plants (Yamamoto et al., 2011) and their lignocellulose yielded almost 50% more glucose per wood weight and per cellulose weight in saccharification after steam pretreatment (Kaida et al., 2009). Similar effects on growth and saccharification were observed in *Acacia mangium* (Kaku et al., 2011; Hartati et al., 2011). However, in a 4-year field trial transgenic poplars expressing *AaXEG2* displayed substantially reduced biomass (Taniguchi et al., 2012). Furthermore, the plants were unable to bend upward when placed horizontally despite forming tension wood as in wild type, suggesting that xyloglucan is essential for generation of tensile stress (Baba et al., 2009).

Suppression of aspen pectin methyl esterase PME1 resulted in highly methylesterified HG in developing wood (Siedlecka et al., 2008; Table 1). The glucose yields in saccharification without pretreatment increased, but there was no improvement after acid pretreatment (Escamez et al., 2017).

Overexpression of aspen wood-expressed pectate lyase PL1-27 increased the solubility of xylan and xyloglucan suggesting that HG constrains the solubility of main non-cellulosic polysaccharides (Biswal et al., 2014; Table 1). There was a positive effect on the glucose yield for the transgenic lines, but only after acid pretreatment. Interestingly, decreased HG biosynthesis in poplar by KD of the *GAUT* genes involved in biosynthesis of pectin and xylan (Mohnen, 2008), led to substantial growth stimulation and a small increase in glucose yields in saccharification after acid or hot-water pretreatment (Biswal et al., 2015; Biswal et al., 2018; Table 1).

## Future Prospects for Improving Non-Cellulosic Polysaccharides for Biorefining of Wood

Two decades of research on modifying non-cellulosic polysaccharides have provided some insight on the role of these polysaccharides in cell wall architecture, on their importance for the efficiency of pretreatment and enzymatic saccharification, and have identified some off-target effects. They have also identified the most promising targets for achieving better growth and saccharification. Most research has been focused on xylan modification identifying xylan chain length, degree of acetylation, glucuronosylation, and glucuronosyl methylation as possible targets. The discovery of microbial enzymes hydrolyzing ester links between glucuronosyl units and lignin opened new prospects for directly reducing LCCs in cell walls, and should be further explored. The modification of minor wood components, HG and xyloglucan, had some of the highest impacts on saccharification, pointing to the crucial role of these polymers in wood integrity, but their modification sometimes led to growth inhibition. Using the wood-specific promoters, such as the *GT43B* promoter (Ratke et al., 2015), for expressing transgenes, can prevent off-target modifications in meristems, root hairs and other primary walled tissues, and possibly avoid growth penalty. Overall, there were few attempts in trees to employ different promoters for expressing the transgenes. Utilization of heat inducible promoters and heat-active enzymes in trees for modifying properties during post-harvest heat treatment has not yet been explored. Modification of other non-cellulosic components, for example mannans, RG-I and RG-II has not been so far investigated in trees and will be interesting to reveal the role of these polymers in woody biomass organization.

Currently, there is little understanding of the molecular mechanisms responsible for the observed phenotypes. In most cases, the distinction between primary and secondary effects of transgene expression is not possible. Interestingly, some types of xylan modification lead to increased growth (Biswal et al., 2015; Derba-Maceluch et al., 2015; Ratke et al., 2018), which might be mediated by the SCW integrity sensing mechanism (Ratke et al., 2018). On the other hand, the transcriptome analyses in *Arabidopsis* mutants impaired in xylan biosynthesis did not reveal any changes indicative of SCW integrity sensing (Faria-Blanc et al., 2018). It would be important to elucidate if such signaling exists and if so, what triggers it, for successful modification of SCW.

Almost all results reviewed here are based on greenhouse experiments. Experience with xyloglucanase-modified poplar (Taniguchi et al., 2012) points to a need for early field experiments to pinpoint possible problems of transgenic lines. Field-grown trees will also provide sufficient biomass for testing pulping properties.

Finally, the tension wood of hardwoods appears to be particularly suitable for saccharification (Brereton et al., 2012; Sawada et al., 2018). Progress in identification of pathways involved in the induction of tension wood (Felten et al., 2018) will make it possible to design strategies to stimulate tension wood formation in normal growth conditions for dedicated biorefinery feedstocks.

## Author Contributions

ED, LJJ, and EJM wrote the paper. MLG prepared the Figure and the Table. All authors read and approved the final submission.

## Conflict of Interest Statement

The authors declare that the research was conducted in the absence of any commercial or financial relationships that could be construed as a potential conflict of interest.

## Abbreviations

AG-II: type II arabinogalactan
CML: compound middle lamella
GGM: galactoglucomannan
G-layer: gelatinous layer
GM: glucomannan
GT: glycosyltransferase family
GX: glucuronoxylan
HG: homogalacturonan
IRX: irregular xylem
KD: knock-down
LCCs: lignin carbohydrate complexes
PCW: primary cell wall
RES: reducing end sequence in xylan
RG-I: rhamnogalacturonan I
RG-II: rhamnogalacturonan II
S/G: syringyl/guaiacyl
SCW: secondary cell wall
Xyl*p*: xylopyranosyl residue
